# The Utility of Frozen Sections in the Evaluation of Clear Margins in Oral Squamous Cell Carcinomas: A Cross-Sectional Study From a Tertiary Care Center

**DOI:** 10.7759/cureus.22308

**Published:** 2022-02-16

**Authors:** Muhammad Wasif, Ainulakbar Mughal, Muntazir Hussain, Mahum Zaidi, Muhammad Sohail Awan, Sabeeh Sidddique, Ozair Awan, Shayan K Ghaloo, Anwar Suahil

**Affiliations:** 1 Otolaryngology - Head and Neck Surgery, Dr. Ziauddin University Hospital, Karachi, PAK; 2 Otolaryngology - Head and Neck Surgery, Aga Khan University Hospital, Karachi, PAK; 3 Head and Neck Surgery, Cancer Foundation Hospital, Karachi, PAK; 4 Diagnostic Radiology, Liaquat National Hospital, Karachi, PAK; 5 Otolaryngology, Aga Khan University Hospital, Karachi, PAK; 6 Pathology, Aga Khan University Hospital, Karachi, PAK; 7 Otolaryngology, Aga Khan University Medical College, Karachi, PAK; 8 Surgery, Aga Khan University Hospital, Karachi, PAK

**Keywords:** head and neck cancer, tumor margins, squamous cell carcinoma, oral cavity, frozen section

## Abstract

Background and objective

Head and neck cancers are prevalent in Pakistan. Oral squamous cell carcinomas are primarily treated via surgical removal, and complete surgical resection is the paramount prognostic factor. A resection margin of 5 mm on the final histopathology report has been accepted as adequate in the existing literature. Negative margins on the frozen section do not guarantee adequate disease-free resections on the final histopathology report. In this study, we aimed to ascertain how accurately tumor-free margins can be detected on frozen sections, which are reported intraoperatively compared to permanent sections of the same tissues reported after proper staining in oral squamous cell carcinoma patients.

Methods

A cross-sectional study was conducted at a tertiary care hospital in Karachi, Pakistan; 94 patients presenting between January and October 2016 were included in this study and a total of 432 tumor margins were assessed.

Results

Among the total 94 patients included in the study, 79% were male and 21% were female. Buccal mucosa was the most commonly involved subsite (57%), followed by the tongue (25%). The most common T stage was T4 (33%), followed by T2 and T3 at 28% and 21% respectively, while the most common N stage was N0 (55%) followed by N1 at 16% and N2 at 22%. The sensitivity of the frozen section in comparison to the permanent section was found to be 50%, while specificity was calculated to be 99.8%. The positive predictive value was 75% and the negative predictive value was 99.3%.

Conclusion

The frozen section is a highly useful tool for the evaluation of tumor margins. However, while it has high diagnostic accuracy rates, it can produce altered results and therefore requires high clinical correlation.

## Introduction

Head and neck cancers are prevalent in Pakistan, accounting for as high as 20% of all cancers in male patients and 10% of all cancers in female patients [1]. Almost 62,000 new patients are reported in the United States every year, with a mortality rate of 13,000 per annum [2], and squamous cell carcinoma is the sixth most common cancer worldwide [2]. The vast majority of tumors of the oral cavity, approximately 90%, are squamous cell carcinomas [3].

Oral squamous cell carcinomas are usually treated by surgical excision [4], the paramount prognostic factor being complete surgical excision as the incomplete removal of the primary tumor frequently leads to poor outcomes [5]. The importance of resection margins in oral cancers is well known, and a resection margin of 5 mm on the final histopathology report has been accepted as adequate in the existing literature [6]. Negative margins on the frozen section do not guarantee adequate disease-free resections on the final histopathology. In their study, Ord et al. showed that the frozen section does not influence the final status of the margin as 10 out of 49 patients in their cohort had positive margins on the final histopathology report [7]. In addition, a prospective study conducted by Yahalom et al. has shown that 40% of tumor margins reported as negative on the frozen section came back as positive on the final histopathology report [8]. Moreover, DU et al. have suggested that negative intraoperative margins do not guarantee clear resections. Although they did report a high sensitivity at 83% and a high specificity at 98%, they also revealed that final positive margins were undetected in 4.3% of frozen sections and close margins were undetected in 17.8% of frozen sections [9].

The inconsistencies in the available data on this topic and the lack of local data on the accuracy of detection of tumor-free margins on frozen sections compared to permanent sections of the same tissues in oral squamous cell carcinoma patients prompted us to conduct a study in our setting. Our objective was to ascertain how accurately tumor-free margins can be detected on frozen sections compared to permanent sections of the same tissues in oral squamous cell carcinoma patients. This study will help head and neck surgeons in devising protocols and guidelines regarding the utilization of frozen sections in oral squamous cell carcinomas.

## Materials and methods

A cross-sectional study was conducted at a tertiary care hospital in Karachi, Pakistan. A total of 94 patients presenting between January and October 2016 were included in this study after obtaining approval from the institutional ethical review committee (ERC no. 4545-Sur-ERC-16). Medical records of these patients were reviewed and data were collected from the online database, which included demographics and clinical data including frozen section results and final permanent histopathologic results of all patients in whom frozen sections were sent intraoperatively for immediate diagnosis. Five different head and neck surgeons performed these resections and the decision to send frozen sections was based on the individual surgeon’s clinical judgment. A total of 432 frozen sections were sent during the treatment of these 94 patients.

Frozen sections were reported by a histopathologist during the surgery as either positive (having microscopic evidence of tumor) or negative (no microscopic tumor); further, the same margins were stained and reported as a part of the permanent section as well, as either positive or negative for tumors. The results of the frozen sections were then compared with the results of the permanent sections to determine the sensitivity and specificity in the detection of tumor-free margins on frozen sections, as well as the positive and negative predictive values.

SPSS Statistics version 19 (IBM, Armonk, NY) was used for statistical analysis. All continuous variables were reported as means and standard deviations whereas categorical variables were presented as percentages and proportions. The accuracy of frozen sections to determine tumor-free margins was ascertained by analyzing the sensitivity and specificity in addition to the positive and negative predictive values.

## Results

The clinicopathologic characteristics of the 94 included patients are listed in Table 1. A total of 432 frozen sections were assessed with an average number of 4.5 frozen sections per case. The mean age of the patient population was 52 years; 79% of the cohort were male and 21% were female. Buccal mucosa was the most commonly involved subsite (57%), followed by the tongue (25%). The most common T stage was T4 (33%), followed by T2 and T3 at 28% and 21% respectively, while the most common N stage was N0 (55%) followed by N1 at 16% and N2 at 22%. Most of our patients presented with an advanced stage disease with stages III and IV observed in 55% of the population (Table 1).

A positive frozen section report warranted further resection till a normal mucosal margin was achieved. A total of four margins were positive on the frozen section out of which one was reported as negative on the permanent paraffin-embedded sections. Out of the 428 margins that were reported to be negative on the frozen section, three were reported as positive on the permanent paraffin-embedded sections.

The overall sensitivity of the frozen section compared to the permanent section was found to be 50% and specificity was found to be 99.8%, whereas the positive and negative predictive values were found to be 75% and 99.3% respectively. The overall diagnostic accuracy was calculated to be 99% (Table 2). Table 1 presents the clinicopathologic characteristics in our cohort.

**Table 1:** Clinicopathologic characteristics of 94 patients with oral cavity squamous cell carcinoma SD: standard deviation

Variables	Number of cases	%
Age, years, mean ± SD	52 ± 12	
Gender		
Male	74	78.7
Female	20	21.3
Subsite		
Buccal	54	57
Tongue	23	25
Others	17	18
Pathologic tumor stage		
I	15	16
II	27	29
III	20	21
IV	32	34
Pathologic N stage		
0	53	56
1	15	16
2	22	24
3	1	1
x	3	3

**Table 2:** 2x2 table showing the comparison between permanent section and frozen section

		Permanent section			
		Positive	Negative	Total	
Frozen section	Positive	3	1	4	
	Negative	3	425	428	
	Total	6	426	432	

## Discussion

Squamous cell carcinomas are among the most common cancers in our region. Primary surgery is the main treatment for oral cancers. The primary aim of surgical excision is to achieve clear margins during the surgery; however, a surgeon’s judgment of clear margins during the surgery can be faulty because of the presence of microscopic disease that can be easily missed by the naked eye. In a study involving 1,237 patients, Datta et al. found that there was microscopic spread beyond the gross disease margins in 8.6% of patients and this led to a modification of the gross margin status in 5.2% of the patients [10]. Thus, frozen sections have an important role as they help pathologists assess margins in microscopic detail and timely inform surgeons while the patient is still in the operating room.

Patients with positive and close (less than 5 mm) margins have a very high chance of local recurrence and consequent poor overall survival, even after adjuvant therapies. Gerber et al. have demonstrated that the five-year overall survival was reported to be 60% in patients who had clear resection margins compared to 52% in patients who had involved resection margins in cases of oral cancers. Additionally, the incidence rate of recurrence was found to be double (36%) in patients who had positive tumor margins as opposed to 18% in patients who had negative tumor margins [11].

The reported accuracy of determining tumor-free margins via frozen sections varies between 96 and 98% in the resection of cancers of the oral cavity. In our study, the overall sensitivity of frozen sections compared to permanent sections was found to be 50% while the specificity was found to be 99.8%. The positive and negative predictive values were 75% and 99.3% respectively, and the overall accuracy of the diagnosis was reported to be 99%. These statistics are comparable to those in the published literature [7,8,12]; however, the low sensitivity might be due to the fact that only six of the samples turned out to be positive on final histopathology.

Three patients with positive margins were correctly identified using frozen sections and hence underwent further surgical removal of tissues. However, there was one patient who was incorrectly reported to have positive margins on the frozen section, which resulted in the unnecessary removal of excess tissue. A total of three patients were found to have false-negative results on frozen section samples as all three of these patients were found to have severe dysplasia on analysis of the paraffin-embedded sections. Thus, these samples were labeled to have positive margins by the histopathologist.

This issue of margin shrinkage in resection of cancers of the oral cavity is often not given much attention. Many times, an adequate tumor margin can become altered and converted to a close resection margin on the final permanent histopathology report due to shrinkage of tissues. Johnson et al. studied this issue of shrinkage of margins in the oral cavity of dogs and reported the tissue shrinkage in the time period from removal during surgery to examination under a microscope to be between 30.7-47.3% [13]. In light of this, their suggestion was to ensure that at least 8-10 mm of healthy tissue was resected in order to achieve a clear resection margin of more than 5 mm [14].

## Conclusions

The frozen section is a highly useful tool for the evaluation of tumor margins. It can help surgeons in deciding whether adequate margins have been taken or not in oral cavity squamous cell carcinomas. Our results have shown that frozen sections have high diagnostic accuracy rates. However, they can produce altered results and hence require high clinical correlation.
